# Catching Latrophilin With Lasso: A Universal Mechanism for Axonal Attraction and Synapse Formation

**DOI:** 10.3389/fnins.2019.00257

**Published:** 2019-03-22

**Authors:** Yuri A. Ushkaryov, Vera Lelianova, Nickolai V. Vysokov

**Affiliations:** ^1^Medway School of Pharmacy, University of Kent, Chatham, United Kingdom; ^2^BrainPatch Ltd., London, United Kingdom

**Keywords:** teneurin, latrophilin, lasso, axonal attraction, cell adhesion

## Abstract

Latrophilin-1 (LPHN1) was isolated as the main high-affinity receptor for α-latrotoxin from black widow spider venom, a powerful presynaptic secretagogue. As an adhesion G-protein-coupled receptor, LPHN1 is cleaved into two fragments, which can behave independently on the cell surface, but re-associate upon binding the toxin. This triggers intracellular signaling that involves the Gαq/phospholipase C/inositol 1,4,5-trisphosphate cascade and an increase in cytosolic Ca^2+^, leading to vesicular exocytosis. Using affinity chromatography on LPHN1, we isolated its endogenous ligand, teneurin-2/Lasso. Both LPHN1 and Ten2/Lasso are expressed early in development and are enriched in neurons. LPHN1 primarily resides in axons, growth cones and presynaptic terminals, while Lasso largely localizes on dendrites. LPHN1 and Ten2/Lasso form a trans-synaptic receptor pair that has both structural and signaling functions. However, Lasso is proteolytically cleaved at multiple sites and its extracellular domain is partially released into the intercellular space, especially during neuronal development, suggesting that soluble Lasso has additional functions. We discovered that the soluble fragment of Lasso can diffuse away and bind to LPHN1 on axonal growth cones, triggering its redistribution on the cell surface and intracellular signaling which leads to local exocytosis. This causes axons to turn in the direction of spatio-temporal Lasso gradients, while LPHN1 knockout blocks this effect. These results suggest that the LPHN1-Ten2/Lasso pair can participate in long- and short-distance axonal guidance and synapse formation.

## Isolation and Architecture of Latrophilin

This story began in the early 1970s, when it was found that the venom from the black widow spider, *Latrodectus mactans*, causes massive release of neurotransmitters from vertebrate synapses (Longenecker et al., 1970). The neurotoxin purified from this venom, α-latrotoxin (αLTX), was shown to form Ca^2+^-permeable pores in artificial membranes (Finkelstein et al., 1976). However, it acted only after binding a high-affinity presynaptic receptor/s in neuronal cells. Even more intriguingly, αLTX could act in the absence of extracellular Ca^2+^ (Longenecker et al., 1970). These findings suggested that the toxin receptor had a potential to stimulate the presynaptic neurotransmitter release machinery directly, bypassing the requirement for Ca^2+^ in vesicular exocytosis.

Fascinated by these characteristics, several groups began their quest for the Ca^2+^-independent αLTX receptor, using the toxin as an affinity adsorbent (Scheer and Meldolesi, 1985; Ushkarev and Grishin, 1986; Petrenko et al., 1990). The first receptor preparation contained several proteins (Petrenko et al., 1990), of which the largest was termed neurexin Iα (Ushkaryov et al., 1992). However, as neurexin required Ca^2+^ to bind αLTX and did not display clear signaling capabilities, the search for the Ca^2+^-independent receptor continued. Eventually, two laboratories simultaneously isolated this protein using αLTX affinity columns and called it latrophilin 1 (LPHN1) (Davletov et al., 1996) or Ca^2+^-independent receptor for αLTX 1 (CIRL1) (Krasnoperov et al., 1996). Its amino acid sequence (Krasnoperov et al., 1997; Lelianova et al., 1997) showed homology to G protein-coupled receptors (GPCRs) of the secretin group.

**FIGURE 1:**
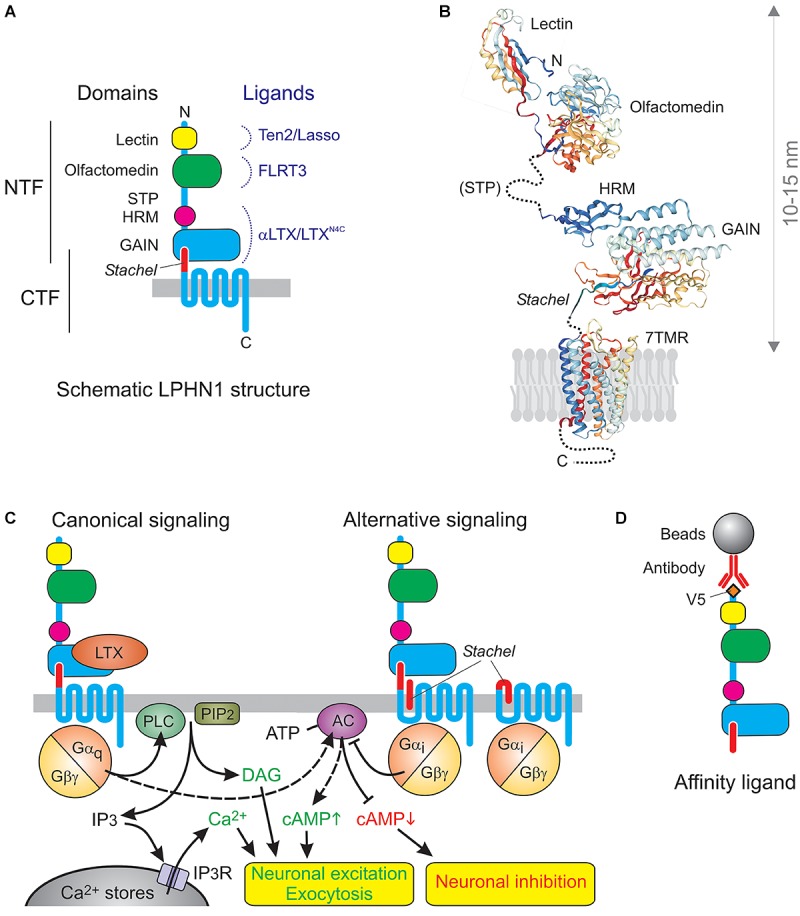
The architecture and signaling functions of LPHN1. **(A)** The domains and ligands of LPHN1. **(B)** 3D structure of LPHN1 domains (Vakonakis et al., 2008; Araç et al., 2012; Ranaivoson et al., 2015). **(C)** Canonical LPHN1 signaling and proposed alternative signaling via the *Stachel* peptide. The expected outcomes of respective signaling pathways are shown below. **(D)** The use of NTF as an affinity adsorbent.

However, the toxin receptor was clearly different (Figure 1A): (1) it had a very long N-terminal extracellular domain (ECD) containing regions of homology to extracellular proteins (lectin and olfactomedin), (2) it was proteolytically cleaved upstream of the first transmembrane domain (TMD), (3) this constitutive cleavage occurred inside the cell and did not lead to signaling (Krasnoperov et al., 2002; Volynski et al., 2004), (4) the resulting N-terminal fragment (NTF) remained largely associated with the 7TMD C-terminal fragment (CTF) (Krasnoperov et al., 1997), but (5) the fragments could dissociate and behave as independent membrane proteins (Volynski et al., 2004; Silva et al., 2009a).

A number of similarly, built receptors was soon identified either biochemically or genetically. Based on their common features, they were isolated into a separate family, “Adhesion GPCRs” (aGPCRs) (Fredriksson, 2003). According to the modern nomenclature recommended by the International Union of Basic and Clinical Pharmacology, the group is now called ADhesion G protein-coupled Receptors (ADGRs), of which LPHN1 represents the Latrophilin subfamily, ADGRL (Hamann et al., 2015).

It is now established that aGPCRs are a large and ancient family of GPCRs (Hamann et al., 2015). They all contain similar 7TMD domains, which also resemble GPCRs from other families, but these are connected to variable C-terminal tails and to a surprisingly vast array of long N-terminal ectodomains. This diversity of the extracellular domain, featuring homology to various protein classes involved in protein-protein interactions and cell-adhesion, combined with a conserved signaling domain, has led to this group being dubbed “chimerical receptors” (e.g., Kwakkenbos et al., 2006), which probably reflects the way they appeared in evolution. In all aGPCRs (except GPR123 with a very short ectodomain) the ectodomains are connected to the 7TMDs by a conserved “GPCR autoproteolysis-inducing” (GAIN) domain (Araç et al., 2012), previously known as a “GPCR proteolysis site” (GPS) (Krasnoperov et al., 1997). The GAIN domain in almost all aGPCRs undergoes internal proteolysis and is then unequally divided between the NTF and CTF: the larger portion of the GAIN domain remains part of the NTF and can bind the smaller C-terminal portion, which forms the very N-terminus of the CTF. This interaction mediates non-covalent association of the fragments (Figure 1A,B), but the two parts of the GAIN domain can dissociate, leading to important changes in receptor functions. This dynamic structure may be key to understanding the physiological functions of aGPCRs. In full agreement with their name, many aGPCRs have been shown to bind large ligands on the surface of other cells or in the extracellular matrix, thus enabling the conversion of extracellular interactions into intracellular signals. Many family members have been demonstrated to signal via G proteins, as proper GPCRs, while others can signal independently of G proteins, however, the signaling capabilities of aGPCRs are only beginning to be understood (Hamann et al., 2015), and LPHN1 is one of the few aGPCRs for which G protein coupling has been unequivocally demonstrated.

## Signaling

LPHN1 signaling has been extensively studied using LTX^N4C^, a mutant αLTX that acts as an exogenous ligand of this receptor but fails to form tetramers and membrane pores (Ichtchenko et al., 1998; Volynski et al., 2003, 2004), which are characteristic of the wild-type αLTX (Orlova et al., 2000). LTX^N4C^ binds to the GAIN domain within the NTF (Krasnoperov et al., 1999; Lin et al., 2004; Araç et al., 2012) with high affinity (∼1 nM) (Ichtchenko et al., 1998; Volynski et al., 2003) and causes a strong and sustained increase in “spontaneous” neurotransmitter release (Ashton et al., 2001; Capogna et al., 2003; Volynski et al., 2004; Lelyanova et al., 2009; Déak et al., 2009). This effect is purely presynaptic, as only the frequency of miniature events is affected, but not their amplitude or duration (Capogna et al., 2003). Unable to make transmembrane pores, LTX^N4C^ can only exert its action via receptor-mediated signaling, and receptor knockout or mutagenesis (leading to a loss of signal transduction) obliterates the toxin-evoked signal (Tobaben et al., 2000; Volynski et al., 2004).

Binding of LTX^N4C^ to the NTF induces its re-association with the CTF and subsequent signaling (Volynski et al., 2004; Silva et al., 2009a; Vysokov et al., 2016, 2018). A very similar behavior was reported also for EMR2 (Huang et al., 2012) and may be a universal feature of all aGPCRs. However, it is not clear whether the NTF-CTF complex has the same structure before the separation of its fragments and after their re-association.

Similar to many other GPCRs, LPHN1 probably activates multiple signaling mechanisms, but at least one that leads to increased neurotransmitter release has been studied in detail (Figure 1C). LTX^N4C^-induced association of the NTF and CTF causes Gαq-mediated (Rahman et al., 1999) activation of phospholipase C (PLC) (Davletov et al., 1998; Capogna et al., 2003; Volynski et al., 2004), which cleaves phosphatidylinositol 4,5-bisphosphate (PIP2), producing inositol 1,4,5-trisphosphate (IP_3_) (Lelianova et al., 1997; Ichtchenko et al., 1998) and diacylglycerol. Both physical and functional interaction of CTF with Gαq was demonstrated by NTF-mediated pull-down experiments, where the CTF–Gαq complex persisted in the presence of GDP, but was lost when GDP was replaced with GTP (Rahman et al., 1999). Furthermore, the overexpression of LPHN1 in COS7 cells itself substantially decreased the resting concentration of IP_3_ (due to non-productive binding of the bulk of cellular Gαq by the inactive overexpressed receptor); reciprocally, activation of LPHN1 upregulated IP_3_ (Lelianova et al., 1997) The specific involvement of Gαq and PLC in LPHN1-mediated effects was experimentally demonstrated in synaptosomes, organotypic neuronal cultures, LPHN1-transfected NB2a cells, LTX-sensitive MIN6 β-cell line, and neuromuscular junctions (Davletov et al., 1998; Capogna et al., 2003; Volynski et al., 2004; Lajus et al., 2006; Lelyanova et al., 2009). The IP_3_-induced increase in cytosolic Ca^2+^ can be inhibited by intracellular Ca^2+^ chelators, intracellular store depletion using thapsigargin, or by inhibition of the IP_3_ receptor using xestospongin C or 2-APB (Davletov et al., 1998; Capogna et al., 2003; Lajus et al., 2006). This demonstrates the strict dependence of LPHN1-mediated effect on intact intracellular Ca^2+^ stores, IP_3_ receptor activity, and ultimately on an increase in cytosolic Ca^2+^ concentration. Calcium released by LTX^N4C^ from the stores is not, however, sufficient to stimulate substantial exocytosis, at least in large synapses, such as neuromuscular junctions (Lelyanova et al., 2009), and extracellular 0.2–1 mM Ca^2+^ is required to support the effect of LTX^N4C^-evoked LPHN1 signaling on neurotransmitter exocytosis (Davletov et al., 1998; Ashton et al., 2001; Capogna et al., 2003; Volynski et al., 2003; Lajus et al., 2006; Lelyanova et al., 2009). This is most likely due to the signaling-induced opening of store-operated Ca^2+^ channels and influx of extracellular Ca^2+^, as hypothesized previously (Ushkaryov et al., 2008). Interestingly, presynaptic Ca^2+^ stores and, more specifically, store-operated Ca^2+^ entry into nerve terminals has been recently shown to play a critical role in the control of neurotransmitter release (de Juan-Sanz et al., 2017).

The endogenous ligand of LPHN1 teneurin-2 (Ten2), or Lasso, (see below) causes a similar NTF-CTF reassociation and rise in cytosolic Ca^2+^ which then stimulates rapid store-operated Ca^2+^ entry (Silva et al., 2011; Vysokov et al., 2018), although the duration of the Ten2/Lasso effect is relatively short (Vysokov et al., 2018). The ligand-bound NTF thus appears to serve as an agonist of the CTF (Volynski et al., 2004; Huang et al., 2012), although the CTF may have its own ligands.

In fact, at least some signaling by free CTF may be induced by the small piece of the ECD that remains at the N-terminus of the CTF after the cleavage of NTF (Figure 1C). This hydrophobic peptide, called 7 amino acids (Volynski et al., 2004), stalk (Kishore et al., 2016) or *Stachel* peptide (Liebscher et al., 2014), can act as a “tethered ligand” (Liebscher et al., 2014; Stoveken et al., 2015). Normally, *Stachel* mediates the interaction between the CTF and NTF. It is thought that conformational changes induced by ligand binding to the NTF (or its complete removal) free up *Stachel* peptide, allowing it to interact with the 7TMD and trigger signaling (Liebscher et al., 2014; Stoveken et al., 2015; Nazarko et al., 2018). Micromolar concentrations of exogenous *Stachel* can activate signaling even without ligand binding to, or removal of, the NTF (Liebscher et al., 2014; Nazarko et al., 2018; Figure 1B). However, in LPHN1 *Stachel*-induced signaling appears to be different from that produced by the binding of NTF ligands. Thus, exogenous *Stachel* peptide caused a pertussis toxin-sensitive decrease in cAMP levels (Nazarko et al., 2018). By contrast, NTF ligands usually increase cAMP levels (Figure 1C, left) [e.g., after activation of LAT-1 by its endogenous ligand in *Caenorhabditis elegans* (Winkler and Prömel, 2016) or activation of rat LPHN1 expressed in COS7 cells by LTX (Lelianova et al., 1997)]. Also, the NTF-CTF complex did not bind Gαi in pull-down experiments (while Gαs was not tested) (Rahman et al., 1999).

These data indicate that LPHN1 might send different intracellular signals depending on (1) the interaction between the NTF and CTF, (2) the agonist involved and (3) the state of cell’s signaling and protein modification machinery.

## Isolation of Lasso

Several features of LPHN1 – (1) the ability of its NTF (in complex with its ligand/s) to activate the CTF (Volynski et al., 2004; Silva et al., 2009a) and send an exocytotic signal; (2) the size of the NTF, which is sufficient to span half of the synaptic cleft; and (3) the presynaptic localization of LPHN1 (Silva et al., 2011; Vysokov et al., 2016) – led us to hypothesize that the NTF could bind a postsynaptic ligand. Not only would then the NTF, being held at the active zone by trans-synaptic interactions with a postsynaptic protein, always localize close to presynaptic vesicle release sites, but it would also provide presynaptic docking sites for the independently recycling CTF and potentially enable retrograde signaling (Volynski et al., 2004). These ideas prompted us to start looking for an LPHN1 ligand, operationally called “LPHN1-associated synaptic surface organizer” (Lasso) (Silva et al., 2009b).

When designing a soluble LPHN1 construct to make an affinity column (Figure 1D), we relied on our knowledge of the NTF-CTF relationship. Thus, although the NTF-CTF complex has a high affinity for αLTX/LTX^N4C^, it can also dissociate (Silva et al., 2009a), possibly upon binding an antagonist, so anchoring the NTF-CTF complex via CTF could be inefficient. On the other hand, if the NTF is synthesized without *Stachel* or if the NTF-CTF cleavage is blocked (e.g., due to a mutation), the NTF assumes a conformation that does not bind αLTX (Silva et al., 2011) but could bind non-specific ligands. Thus we anchored the full ECD (containing the NTF and *Stachel* peptide) on the column via an N-terminal V5 epitope (Figure 1D).

Affinity chromatography of solubilized rat brain on this adsorbent at moderate stringency (0.5 M NaCl), resulted in the isolation of the long-sought Lasso, a protein of ∼270 kDa (Silva et al., 2011). We did not observe even minute amounts of FLRT3 or neurexin, the other proposed ligands of LPHN1 (Boucard et al., 2014; O’Sullivan et al., 2014). This indicates that the chromatography conditions were too stringent for their binding to LPHN1 and that Lasso is the strongest ligand of LPHN1. Subsequent sequencing of highly purified Lasso (Silva et al., 2011) indicated that it was identical to Ten2 (Oohashi et al., 1999).

## Interaction Between Lphn1 and Lasso

**FIGURE 2:**
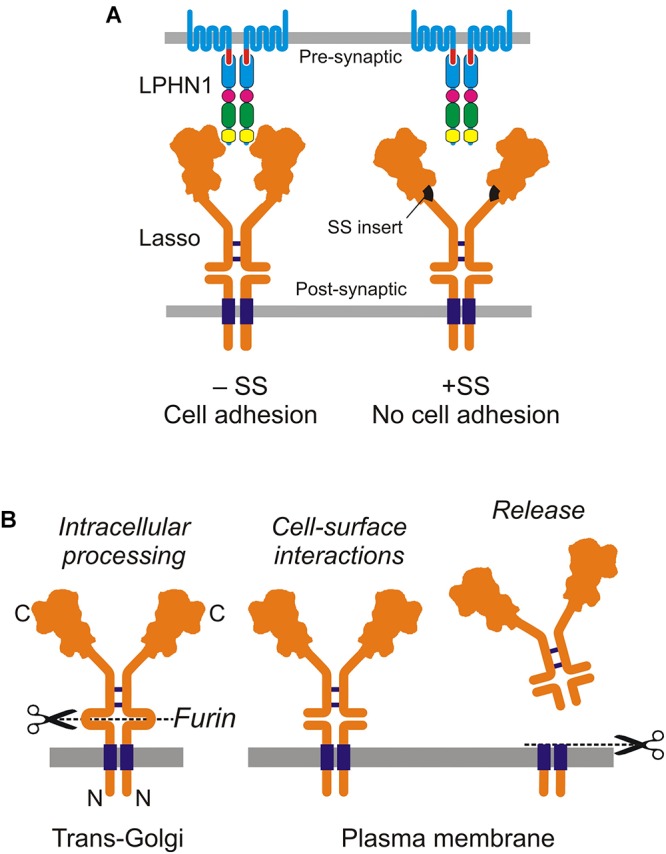
Cell-surface and soluble Ten2/Lasso. **(A)** Cell-surface interactions between LPHN1 and splice variants of Lasso in cell adhesion. **(B)** Cellular processing and release of the soluble ECD of Lasso (from Vysokov et al., 2018).

Ten2/Lasso has a high affinity for LPHN1: the Kd of this complex is 0.47–1.7 nM (Silva et al., 2011; Boucard et al., 2014). The interaction between LPHN1 and Ten2/Lasso is mainly mediated by the lectin-like domain in the NTF of LPHN1 and the C-terminus of Ten2 (Boucard et al., 2014). More narrowly, it involves a short portion of the toxin-like domain of Ten2 that protrudes from the globule (Li et al., 2018). However, we found that this minimal interaction is relatively weak (Silva et al., 2011), but becomes much stronger when other parts of both ECDs are present, especially when Ten2/Lasso constructs are able to dimerize (Silva et al., 2011; Vysokov et al., 2016). Indeed, our observations suggest that dimeric Ten2/Lasso can clasp LPHN1. This could explain why a splice site (SS) in the Ten2 β-propeller domain, which is located far from the toxin-like domain, affects cell-surface interactions between Ten2 and LPHN1 (Li et al., 2018): the small SS insert could change the relative positions of the Ten2 monomers in the dimer, rendering them unable to clasp the LPHN1 molecule (Figure 2A).

The length of the NTF of LPHN1 (as indicated by the crystal or NMR structure of its domains, Figure 1B) is 10–15 nm, while Ten2 is longer than 12 nm (Li et al., 2018), which is sufficient for the two proteins to interact across the synaptic cleft (about 20 nm).

As mentioned, Ten2/Lasso binding to LPHN1 stimulates Ca^2+^ signaling (Silva et al., 2011; Vysokov et al., 2016, 2018; Figure 1C). This is true of the whole soluble ECD of Lasso (Vysokov et al., 2016, 2018) or even its C-terminal toxin-like fragment, when used at higher concentrations (Silva et al., 2011). Furthermore, when Lasso is allowed to interact with LPHN1 prior to LTX^N4C^, it substantially decreases the delay that normally precedes toxin’s action (Vysokov et al., 2018), thought to be required for NTF and CTF rearrangement on the cell surface prior to signaling (Volynski et al., 2004).

## Localization of Lphn1 and Lasso in the Brain

Both LPHN1 and Ten2/Lasso are expressed early in development (Vysokov et al., 2018) and are highly enriched in the CNS, but there seems to be some disagreement regarding the localization of LPHN1 in the synapse. Although LPHN1-mediated effects of α-LTX are irrefutably presynaptic, there have been suggestions that LPHN1 is expressed on the postsynaptic membrane (Meza-Aguilar and Boucard, 2014). This assumption is based on proteome analysis of postsynaptic densities (Collins et al., 2006) and on LPHN1 interaction with a postsynaptic protein Shank3 (Tobaben et al., 2000).

However, these indirect findings did not indicate that LPHN1 was located in the postsynaptic membrane. First, the proteomic study (Collins et al., 2006) only isolated synaptic densities and made no attempt to separate them from presynaptic components tightly associated with postsynaptic components by trans-synaptic complexes and scaffold proteins (Dresbach et al., 2001). As a result, such presynaptic/vesicular proteins as synapsin-1, Munc-13, NSF, bassoon, synaptotagmin-1, and SNAP-25 co-purified with postsynaptic densities even to a greater extent than LPHN1. In contrast, postsynaptic neuroligin appeared to be equally “presynaptic” as its presynaptic ligand neurexin. In addition, it is important to note that the NTF of LPHN1 is non-covalently anchored in the presynaptic membrane and, being strongly bound to Ten2/Lasso on the postsynaptic membrane (Silva et al., 2011), it could ectopically co-purify with postsynaptic membrane. Finally, although the CTF of LPHN1 can interact with Shank3 (Ponna et al., 2018), Shank3 is not exclusively postsynaptic and is also present in presynaptic nerve terminals (Halbedl et al., 2016).

On the other hand, the presynaptic localization of LPHN1 is supported by several findings: during neuronal development LPHN1 concentrates at the leading edge of axonal growth cones (Vysokov et al., 2018) and subsequently becomes enriched in mature nerve terminals (Silva et al., 2011). Furthermore, comparative distribution of Ten2 and LPHN1 in the cerebellum leads to unequivocal conclusions.

Thus, Ten2/Lasso protein is most abundant in the molecular layer of the cerebellum (Zhou et al., 2003). In this layer, the bulk of presynaptic components are provided by granule cell axons (parallel fibers), while the majority of postsynaptic components is located on the dendritic trees of Purkinje and basket cells. Interestingly, Ten2 mRNA is highly expressed in Purkinje, basket and stellate cells, but is almost absent from granule cells (Zhou et al., 2003). LPHN1 protein is also highly enriched in the molecular layer, as evidenced by Ca^2+^-independent α-LTX binding (Davletov et al., 1998). In contrast to Ten2, LPHN1 mRNA is predominantly found in granule cells, but not in Purkinje cells (Lein et al., 2007) and so can only be delivered to the molecular layer with parallel fibers. This complementary expression of the two proteins in the cerebellum strongly indicates that LPHN1 is presynaptic and Ten2/Lasso is postsynaptic, and that they interact across the synaptic cleft. Moreover, this arrangement holds for the bulk of central synapses, as was shown by denaturing synaptic cleft complexes with urea and dithiothreitol and separating pre- and postsynaptic components using differential centrifugation (Berninghausen et al., 2007). After this procedure, 88 ± 8% of the NTF of LPHN1 were clearly presynaptic, while only 12 ± 4% of it might be actually present in the postsynaptic membrane (Silva et al., 2011).

## Cleavage and Shedding of Lasso

Soon after the discovery of Ten2, it was shown to be cleaved at an extracellular furin site between the TMD and EGF repeats (Oohashi et al., 1999; Rubin et al., 1999). This led to suggestions that teneurins can act both as cell-surface receptors and as diffusible signaling molecules (Rubin et al., 1999; Tucker et al., 2001). Furin-induced cleavage was thought to release the ECD into the medium, but it was unclear whether this shedding was constitutive or signaling-induced. Unexpectedly, our recent work showed that furin-mediated proteolysis of Ten2/Lasso occurs constitutively inside the cell (Figure 2B). When this fully cleaved protein is delivered to the cell surface, its ECD remains tethered to the membrane by non-covalent interactions with the fragment containing the TMD (Vysokov et al., 2016).

The shedding of Ten2/Lasso occurs as a result of further, regulated proteolysis at another, near-membrane site, which releases the whole ECD into the medium (Figure 2B). Given that Ten2/Lasso shedding begins early in neuronal cultures (Vysokov et al., 2018), when it is not yet involved in trans-synaptic interactions, and because this shedding slows down dramatically at the end of synaptogenesis (Vysokov et al., 2016, 2018), we thought that Ten2/Lasso cleavage had a role in synapse formation.

What could be the target of released Ten2/Lasso? Homophilic interaction between Ten dimers was previously proposed (Oohashi et al., 1999), and homophilic adhesion between cells expressing exogenous Ten2 was reported (Rubin et al., 2002; Beckmann et al., 2013), but not confirmed by other researchers (Silva et al., 2011; Boucard et al., 2014; Li et al., 2018; Vysokov et al., 2018). On the other hand, we observed a reliable and strong binding of shed Ten2/Lasso to LPHN1 on the surface of cultured cells and axonal growth cones (Vysokov et al., 2016, 2018). This led us to hypothesize (Vysokov et al., 2016) that during neuronal development released ECD of Ten2 could act as a soluble ligand of LPHN1, leading to changes in growth cone behavior.

## Lasso and Latrophilin in Axonal Attraction

As we began exploring the role of LPHN1—Ten2 (-SS)/Lasso interaction in brain development and neurotransmitter release, a series of studies was published describing the role of teneurins in axon guidance (Kenzelmann et al., 2007; Young and Leamey, 2009). This was further confirmed when experiments with Ten3 and Ten2 knockouts in mice demonstrated a profound deficit in at least the visual circuitry (Leamey et al., 2007; Young et al., 2013). However, axon guidance was unlikely to be mediated by the proposed homophilic interactions of Ten2, as they had been shown to inhibit, rather than promote, neurite outgrowth (Beckmann et al., 2013; Young et al., 2013). In addition, symmetric homophilic interactions between teneurins were unlikely to determine the distinct behaviors of axons and dendrites. Therefore, when we discovered that Ten2/Lasso ECD binds LPHN1 (Silva et al., 2011; Vysokov et al., 2016), this suggested fundamentally novel functions for both proteins.

**FIGURE 3:**
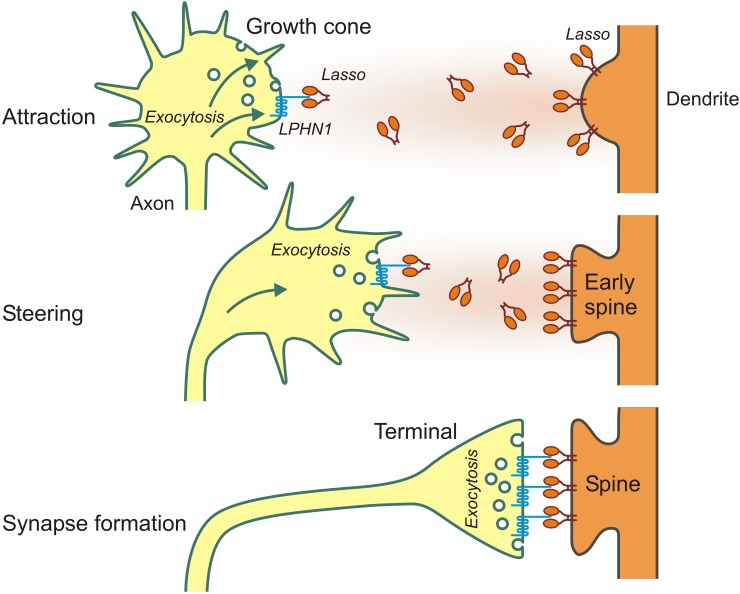
Long- and short-distance interactions of Ten2/Lasso and LPHN1 in axonal attraction and synapse formation.

First evidence to support the role of LPHN1—Ten2/Lasso interaction in axon guidance came from our finding that, in contrast to Lasso, LPHN1 is expressed on axonal growth cones (Vysokov et al., 2018). Additionally, LPHN1 activation by exogenous ligands was known to induce exocytosis via IP_3_-induced Ca^2+^ release (Capogna et al., 2003; Volynski et al., 2003), a mechanism common for many axonal attractants (Tojima et al., 2011). Therefore, it was reasonable to hypothesize that the released fragment of Ten2/Lasso could mediate axonal attraction via LPHN1. We then used microfluidic devices to create spatio-temporal gradients of soluble Ten2/Lasso ECD and demonstrated that attracted rat hippocampal axons, without increasing their general length (Vysokov et al., 2018; Figure 3). Importantly, this steering effect was mediated by LPHN1, because it was not detected in LPHN1 knockout mice.

We also demonstrated a possible mechanism for this attraction, whereby released ECD of Ten2/Lasso, similar to LTX^N4C^, was able to bind LPHN1 on transfected cells and growth cones, causing an association of LPHN1 fragments, induction of Ca^2+^ release and an increase in the rate of exocytosis. Again, LPHN1 knockout experiments indicated that LPHN1 is required for such a mechanism (Vysokov et al., 2018).

This mechanism could mediate axonal attraction throughout the CNS, but may not be limited to it. Given that Ten2 is expressed in chicken embryo both in the CNS, but also in dorsomedial edges of somites, craniofacial mesenchyme and developing limb buds (Tucker et al., 2001), it is tempting to speculate that Ten2/Lasso released by peripheral tissues could also serve as a diffusible factor attracting motor and sensory axons to grow toward their peripheral targets.

Taken together, these results indicate that the shed ECD of Lasso/Ten2 can act as a soluble guidance molecule through its interaction with LPHN1. This work has provided a plausible first explanation of teneurins’ role in brain development and discovered a universal mechanism that uses the same protein-protein interactions both for long-distance axonal attraction and for cell contacts during synapse formation (as summarized in Figure 3).

## Author Contributions

YU conceived and coordinated the work, wrote the manuscript. VL and NV analyzed the literature and wrote parts of the manuscript. All authors contributed to the conception and/or writing of the manuscript.

## Conflict of Interest Statement

NV is affiliated with BrainPatch Ltd. The remaining authors declare that the research was conducted in the absence of any commercial or financial relationships that could be construed as a potential conflict of interest.
